# Deep-Learning Approach to Predict Survival Outcomes Using Wearable Actigraphy Device Among End-Stage Cancer Patients

**DOI:** 10.3389/fpubh.2021.730150

**Published:** 2021-12-09

**Authors:** Tien Yun Yang, Pin-Yu Kuo, Yaoru Huang, Hsiao-Wei Lin, Shwetambara Malwade, Long-Sheng Lu, Lung-Wen Tsai, Shabbir Syed-Abdul, Chia-Wei Sun, Jeng-Fong Chiou

**Affiliations:** ^1^School of Medicine, College of Medicine, Taipei Medical University, Taipei, Taiwan; ^2^Biomedical Optical Imaging Lab, Department of Photonics, College of Electrical and Computer Engineering, National Yang Ming Chiao Tung University, Hsinchu, Taiwan; ^3^Department of Hospice and Palliative Care, Taipei Medical University Hospital, Taipei, Taiwan; ^4^Department of Radiation Oncology, Taipei Medical University Hospital, Taipei, Taiwan; ^5^Graduate Institute of Biomedical Materials and Tissue Engineering, College of Biomedical Engineering, Taipei Medical University, Taipei, Taiwan; ^6^International Center for Health Information Technology, College of Medical Science and Technology, Taipei Medical University, Taipei, Taiwan; ^7^Clinical Research Center, Taipei Medical University Hospital, Taipei, Taiwan; ^8^TMU Research Center of Cancer Translational Medicine, Taipei Medical University, Taipei, Taiwan; ^9^Department of Medical Research, Taipei Medical University Hospital, Taipei, Taiwan; ^10^Graduate Institute of Biomedical Informatics, College of Medical Science and Technology, Taipei Medical University, Taipei, Taiwan; ^11^School of Gerontology and Health Management, College of Nursing, Taipei Medical University, Taipei, Taiwan; ^12^Department of Radiology, School of Medicine, College of Medicine, Taipei Medical University, Taipei, Taiwan

**Keywords:** palliative care, performance status, survival prediction, prognostic accuracy, wearable technology, deep learning, long short-term memory networks, actigraphy

## Abstract

Survival prediction is highly valued in end-of-life care clinical practice, and patient performance status evaluation stands as a predominant component in survival prognostication. While current performance status evaluation tools are limited to their subjective nature, the advent of wearable technology enables continual recordings of patients' activity and has the potential to measure performance status objectively. We hypothesize that wristband actigraphy monitoring devices can predict in-hospital death of end-stage cancer patients during the time of their hospital admissions. The objective of this study was to train and validate a long short-term memory (LSTM) deep-learning prediction model based on activity data of wearable actigraphy devices. The study recruited 60 end-stage cancer patients in a hospice care unit, with 28 deaths and 32 discharged in stable condition at the end of their hospital stay. The standard Karnofsky Performance Status score had an overall prognostic accuracy of 0.83. The LSTM prediction model based on patients' continual actigraphy monitoring had an overall prognostic accuracy of 0.83. Furthermore, the model performance improved with longer input data length up to 48 h. In conclusion, our research suggests the potential feasibility of wristband actigraphy to predict end-of-life admission outcomes in palliative care for end-stage cancer patients.

**Clinical Trial Registration:** The study protocol was registered on ClinicalTrials.gov (ID: NCT04883879).

## Introduction

Accurate survival prediction is highly valued in the clinical practice of end-of-life care. It enables better communication and preparation for impending death, helps avoid futile medical treatment, and facilitates optimal palliative care quality for patients, families, and physicians altogether (1–3). Several validated prognostic tools are available, including Palliative Prognostic Score (PaP) (4–6), Palliative Prognostic Index (PPI) (7–9), Prognosis in Palliative care study (PiPS) score (10, 11), and Glasgow Prognostic Score (12, 13). These scoring systems employ a combination of subjective clinical parameters and/or objective biomarkers to generate survival predictions. Among the parameters used by these prognostic tools, the evaluation of patient performance status (PS) stands as a predominant component. Commonly used PS assessment tools include Karnofsky Performance Status (KPS) (14), Eastern Cooperative Oncology Group (ECOG) Performance Status (15), and Palliative Performance Scale (PPS) (16). However, applications of these evaluation tools are subjective in nature and require trained healthcare professionals for assessments. These characteristics inevitably lead to issues including intraobserver or interobserver variability (17, 18), overestimating or underestimating (19), discontinuous evaluations of activity status, as well as inconvenient implementation in contexts without healthcare professionals.

With the advent of wearable activity monitoring technology, we are now granted convenient and objective methods for the evaluation of patient functional status. Wearable monitors also enable constant documentation of a patient's activity status which could be retrospectively examined and validated. Because of these benefits, monitoring technologies have been applied in different research areas and yielded valuable information on the relationship between activity status and diseases in clinical fields of gynecology (20), surgery (21), pulmonary (22), nephrology (23), and psychology (24). In addition, a study by Gresham et al. also applied objective PS evaluation in a group of advanced cancer patients, which identified correlations between objective activity data of patients and clinical outcomes of adverse events, hospitalization, and overall survival (25). However, no previous study had employed objective PS data for survival prognostication.

In this study, wearable actigraphy devices were applied in a group of end-stage cancer patients for objective measurement of their activity status. We hypothesized that the objective activity data recorded by the wearable devices contained information to help predict in-hospital death of end-stage cancer patients on their hospital admissions. A deep-learning-based prediction model was developed to analyze activity data and suggest survival outcomes of patients. Furthermore, the prognostic accuracy of the proposed activity monitoring and survival prediction model was compared to a current PS evaluation tool, KPS, and a complex prognostic tool, PPI. Finally, we explored and described the applicability, potential, and limitations of the objective activity data recorded by wearable devices as a simple prognostic parameter in clinical settings.

## Materials and Methods

### Study Setting, Participants, and Procedures

The study was conducted in the hospice care unit of Taipei Medical University Hospital (TMUH) from December 2019 to December 2020. Patients with terminal illnesses were admitted to the unit for palliative care and management of pain and other symptoms. Participants aged > 20 years who had at least one diagnosis of end-stage solid tumor diseases and consented to receive hospice care were recruited. Patients with diagnoses of leukemia or carcinoma of unknown primary, patients with evident signs of approaching death upon admission, patients with no vital signs upon admission, or patients who continued to receive aggressive treatment were excluded from this study. After admission to the hospice care unit, patients and their caregivers were first visited and assessed by registered hospice specialist doctors and nurses. If the patient met the criteria mentioned above, they would be invited to participate in the study. Participants would only be recruited once the informed consent was signed by themselves or their legally authorized representative. The study was approved by the ethical committee of the Taipei Medical University-Joint Institutional Review Board (TMU-JIRB No. N201910041).

Clinical data including age, gender, diagnosis, and comorbidities were collected after successful recruitment. Patients were asked to wear a wristband actigraphy device on their hands without intravenous lines. The wearable actigraphy devices (model no. XB40ACT, K&Y lab, Taipei, Taiwan) used in this study is a tiny gadget that weighs 7 g with dimensions of 44^*^19^*^8 mm and has been previously validated (26) and applied in a sleep quality study among cancer patients (27). The monitor collects three-dimensional data of gravitational acceleration, angular change, and spin change of the patient's hand motion every second and transforms them into three statistical parameters: physical activity, angle, and spin. Participants were instructed to wear the devices throughout their hospital stay except showering time because they were not water-resistant. The information was also forwarded to their caregivers.

Subsequently, subjective PS assessments using the KPS and prognostic evaluations using the PPI were done by two trained specialists. The KPS system is an established tool designed for PS evaluation. The score collaboratively takes ambulation, activity, evidence of disease, self-care, the requirement of assistance, and progression of disease into consideration with a scale that ranges from normal activity (100) to death (0) (14). In addition to PS assessments, we applied a complex prognostic tool based on evaluations of PS and other clinical symptoms, namely PPI, starting from July 16, 2020. We were only able to conduct the PPI assessments due to the participation of an additional specialist, who undertook extra work derived from evaluations of patients' clinical symptoms. PPI considers PS and clinical symptoms of oral intake, edema, dyspnea at rest, and delirium, to generate an overall prognostication. According to the original study, the results range from 0 to 15, and a PPI > 6.0 estimates a survival time of fewer than 3 weeks (7). The same group of specialists conducted all KPS and PPI assessments to ensure interpersonal consistency. After the initial consultation, patient activity data recorded by the actigraphy devices would be synced and uploaded every 2–3 days until the patient was discharged from the hospital. Survival outcomes were documented as either death or discharged in stable condition at the end of each patient's hospital stay.

### Data Pre-processing and LSTM-Based Deep Learning Model

The data collected by the actigraphy device is a time series with three features: physical activity, angle, and spin. The issue of variations in each patient's data length was managed by zero paddings until the maximum length of the time series was reached. To avoid vanishing gradients in the deep learning model, we opted for an average value of 20 timesteps and shortened the time series to <500 timesteps.

In this study, we trained a long short-term memory (LSTM)-based deep learning model to predict the clinical status of patients at discharge, which was either death or discharged in stable condition. Recurrent neural networks (RNN) is a deep learning method well-suited to deal with time series structure (28, 29). However, the vanishing gradient problem of RNN made the tool suboptimal for long time-series data (30) for which the LSTM, a particular type of RNN, was used to resolve the issue. Compared to RNN, the LSTM architecture is more resistant to vanishing gradients and allows robust processing of long time-series data (31, 32). The performance of LSTM has been validated in disciplines of economic, financial, stock market forecasting, and even stress forecasting using survey data and physiology parameters. In these studies, LSTM demonstrated lower error rates (33), lower variance (34), and higher accuracy (34, 35) than other analytical methods. A study by Umematsu et al. also showed that LSTM could generate satisfactory results based on objective data measured by wearable devices and phones (35).

**Figure 1:**
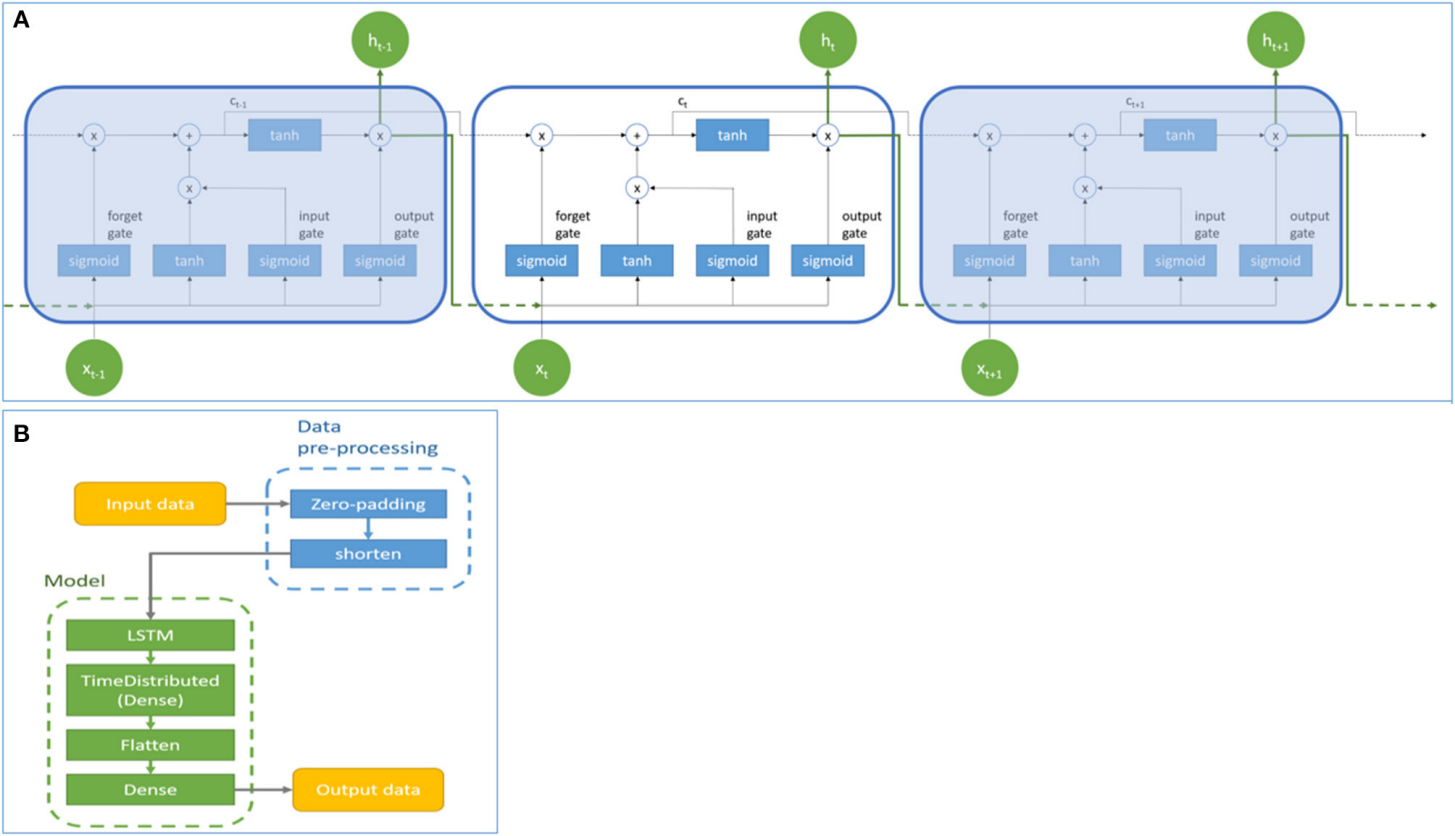
The basic architecture **(A)** and data pre-processing and architectural flow **(B)** of the Long Short-Term Memory model. Symbol x and h represent the input and output values of the LSTM cell. Symbol c represents the value of the memory cell in each LSTM cell. Subscript t represents the time step.

Figure 1A showed the basic architecture of the LSTM model. Symbol x and h represent the input value and the output value of the LSTM cell, respectively. The value in the memory cell in each LSTM cell is c. The subscripts of x, h, and c represented different time points. Each LSTM cell contains an input gate, forget gate, and output gate. The input gate determines whether the neuron writes input values into the memory cell. The forget gate determines whether the memory cell formats memory values. The output gate determines whether the neuron reads the values in the memory cell. The hyperbolic tangent function (tanh) and sigmoid function (σ) are activating functions in LSTM. In this study, the prediction model was based on the LSTM cell to process the three-dimensional time-series data. Data pre-processing and model architecture flows were presented in Figure 1B. The model consisted of an LSTM layer, a dense layer wrapped with TimeDistributed, a flatten layer, and a dense layer. Parameters were adjusted according to different model structures and are presented in the results section. It should be noted that the model was designed to generate survival predictions based solely on activity data of patients, therefore, demographic and clinical data of patients (such as comorbidities) were not utilized by the model.

### Statistical Analysis

Patient characteristics were summarized using descriptive statistics. The clinical outcomes of participants were determined at the end of their hospital stay as binary results: death (1) or discharged in stable condition (0). We adopted a validated cutoff value of 50% for KPS (36) and a cutoff value of 6.0 for PPI as suggested by the original study (7). A receiver operating characteristic (ROC) curve analysis was also conducted to identify optimal cutoff values based on our dataset. The predictive accuracy of KPS and PPI were presented as sensitivity, specificity, positive predictive value (PPV), negative predictive value (NPV), overall accuracy, and the area under the receiver operating characteristic (ROC) curve (AUC). Additionally, an exploratory analysis was conducted to investigate the predictive correlation between KPS and the LSTM model. Correlation between the two variables was calculated using the Pearson correlation coefficient. Statistical analyses were computed using Python version 3.6 and R software version 4.0.2.

## Results

### Demographics of Study Population

From December 11, 2019, to December 10, 2020, 60 patients admitted to the hospice care unit of TMUH were eligible for study recruitment and consented to participate. Patient characteristics, information on KPS and PPI, and their clinical outcomes at discharge were presented in Table 1. The mean age was 72.9 years old (SD 12.2), and 62% were male. Gastrointestinal tumors were the most common malignancies, followed by lung, genitourinary, gynecological, breast, head and neck, and CNS cancers. Seventy seven percent of participants had one or more comorbidities, consisting of hypertension, diabetes mellitus, hyperlipidemia, coronary artery diseases, cerebral infarctions, and others. The median length of hospital stay of patients was 10 (IQR 5-15) days. Twenty eight (47%) patients died at the end of their hospice care stay, whereas 32 (53%) patients were discharged from the hospice care unit in stable condition. It should be noted that one case was discharged against medical advice and deemed as discharged in stable condition. KPS assessments were available or 59 participants, with 28 of them having a KPS score < 50% at admission. PPI assessments were available for 20 participants, with 8 of them having a PPI score > 6.0 on admission.

**Table 1:** Patient demographics and characteristics at baseline visit.

**Characteristics (*N* = 60)**	**Value**	
**Age, years**		
• Mean	72.9	
• SD	12.2	
• Range	45–94	
**Sex**, ***N*** **(%)**		
• Male	37 (61.67%)	
• Female	23 (38.33%)	
**Primary tumor site**, ***N*** **(%)**		
• Gastrointestinal system	26 (43.33%)	
• Lung	12 (20.00%)	
• Genitourinary system	10 (16.67%)	
• Gynecological system	5 (8.33%)	
• Breast	3 (5%)	
• Head and neck	2 (3.33%)	
• Central nervous system	2 (3.33%)	
Patients with comorbidities, *N* (%)	46 (76.67%)	
**Length of hospital stay, days**		
• Median (IQR)	10 (5–15)	
**Patient status at discharge**		
• Death	28 (46.67%)	
• In stable condition	32 (53.33%)	
**KPS (*****N*** **= 59)**	Death	Discharged in stable condition
• KPS < 50%	23 (38.98%)	5 (8.47%)
• KPS ≥ 50%	5 (8.47%)	26 (44.07%)
**PPI (*****N*** **= 20)**	Death	Discharged in stable condition
• PPI > 6.0	8 (40.00%)	0 (0.00%)
• PPI ≤ 6.0	1 (5.00%)	11(55.00%)

### Prognostic Accuracy of KPS and PPI

**Figure 2:**
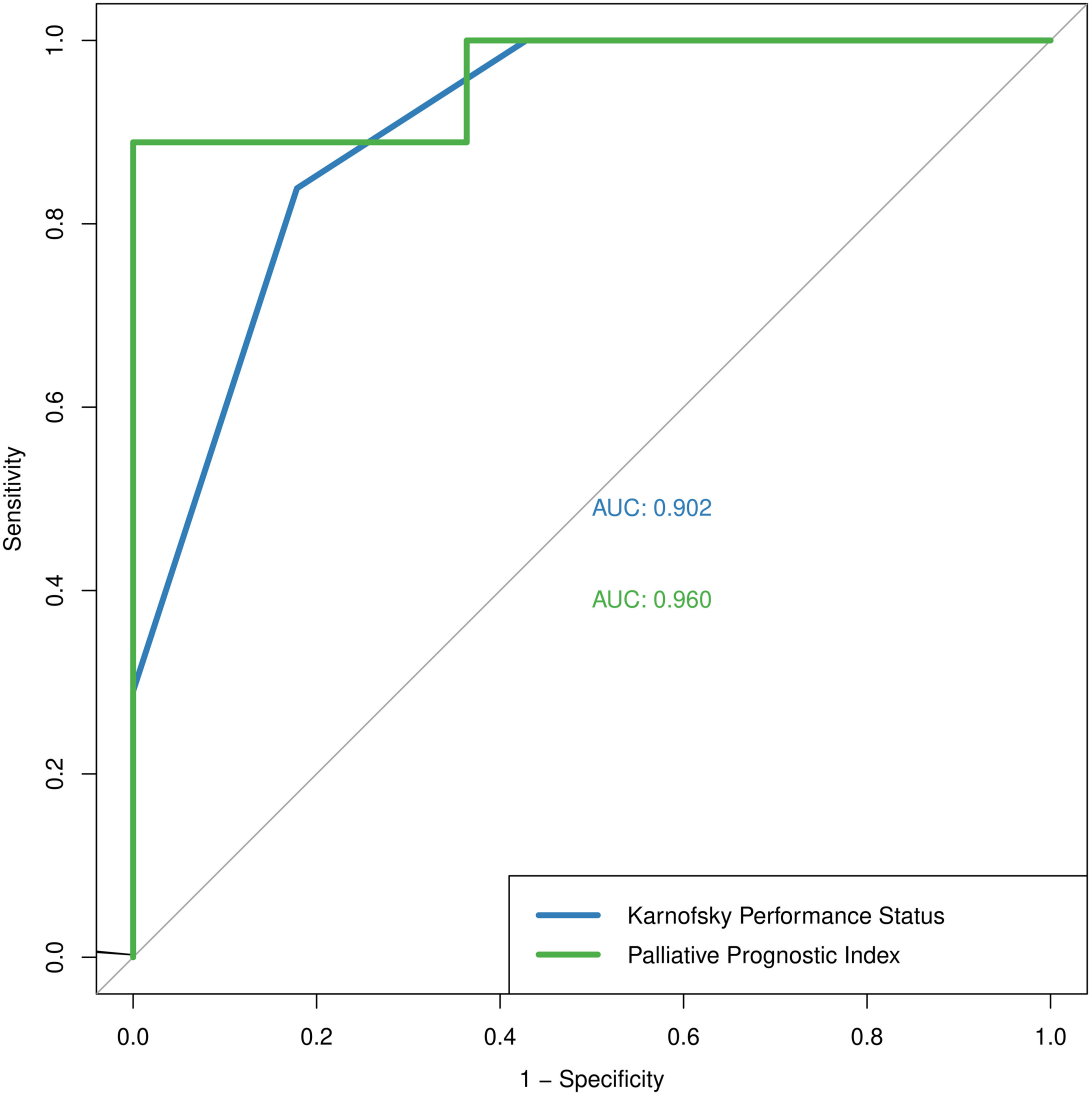
The Receiver Operating Characteristic curve of Karnofsky Performance Status (blue) and Palliative Prognostic Index (green).

The absolute numbers of the true positive, false positive, false negative, and true negative of KPS and PPI assessments are presented in Table 1. True positive was defined as participants with KPS < 50% or PPI > 6.0 at baseline visit and death at the end of their hospital stay. The predictive performance of KPS score based on binary outcomes had an overall predictive accuracy of 83.1% (95% CI 71.0–91.6%), sensitivity of 82.1% (95% CI 63.1–93.9%), specificity of 83.9% (95% CI 66.3–94.5%), PPV of 82.1% (95% CI 63.1–93.9%), NPV of 83.9% (95% CI 66.3–94.5%), and AUC of 0.902. The predictive performance of PPI score based on binary outcomes had an overall predictive accuracy of 95.0% (95% CI 75.1–99.9%), sensitivity of 88.9% (95% CI 51.8–99.7%), specificity of 100% (95% CI 71.5–100%), PPV of 100% (95% CI 63.1–100%), NPV of 91.7 (95% CI 61.5–99.8%), and AUC of 0.960. The discrimination thresholds identified by the ROC curve analysis correlated with the cutoff values we initially adopted for both KPS and PPI (Figure 2).

### Activity Dataset Description and Splitting

**Figure 3:**
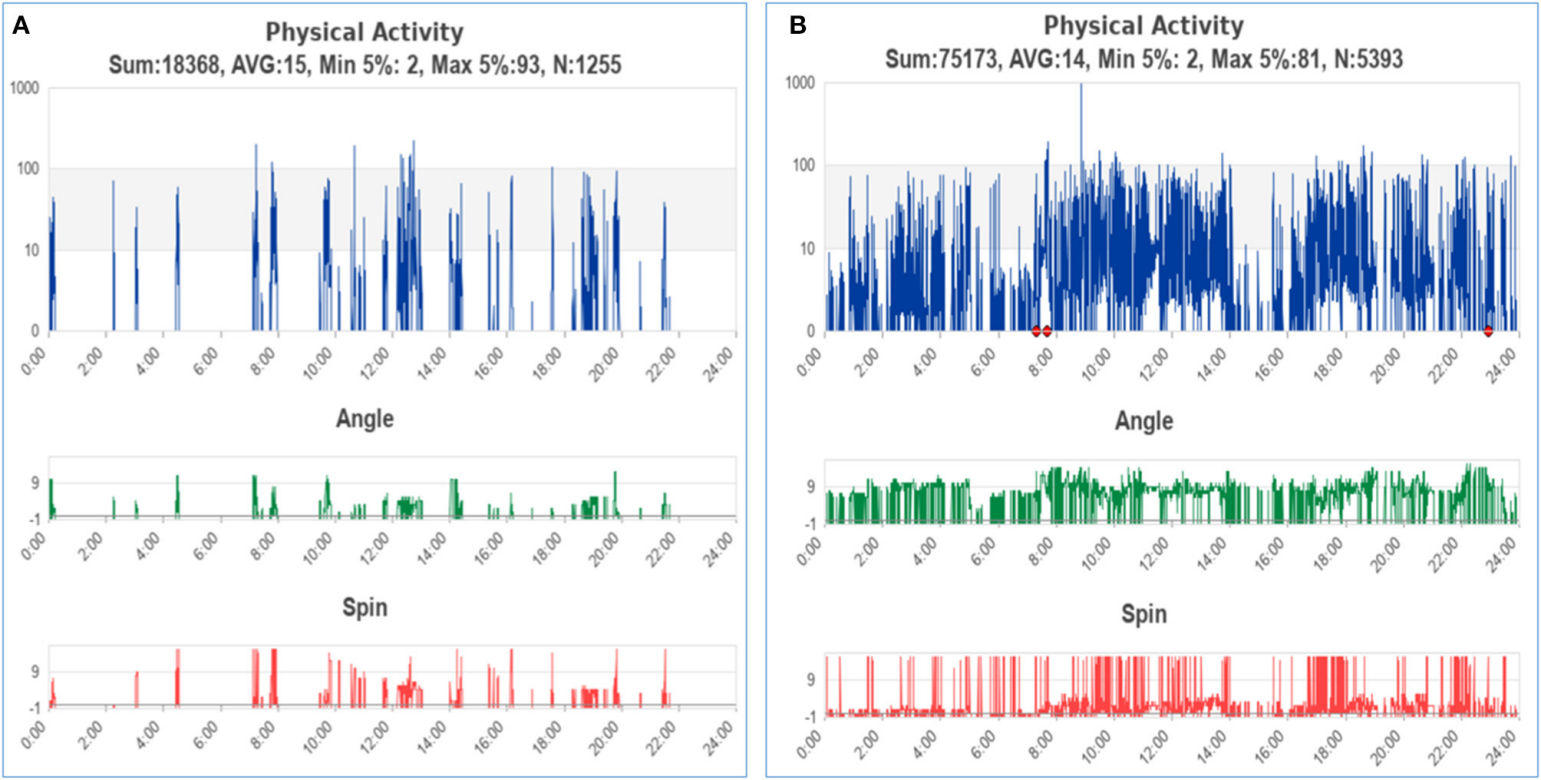
The representative activity pattern of patients with clinical outcomes of death **(A)** and discharged in stable condition **(B)**. The red points on the graph indicated that the patient had taken off the device.

The representative activity pattern recorded by the wearable wristband was shown in Figure 3. Figure 3A belonged to a participant who died at the end of the hospital stay, while Figure 3B belonged to a participant who was discharged in stable condition. Although activity data of patients were recorded throughout their hospital stay, the LSTM-based prediction model only employed data of the initial 48 h for prognostic applicability in clinical settings. After excluding recordings with tracking interruption or data volume of fewer than 48 h, the final dataset included activity data of 44 participants, with 21 deaths and 23 discharged in stable condition at the end of hospital stay, respectively. The maximum length of data after zero-padding is 9,640.

All data was fed into the model after data pre-processing; thus, sampling rates and strides were not defined. We first conducted a preliminary analysis to investigate the feasibility and performance of the model. In the preliminary analysis, the data were divided into a training dataset and a testing dataset at a ratio of 7:3. The number of LSTM units for the preliminary model is 64, with a batch size of 8. We further divided data into training, validation, and testing datasets at a ratio of 7:2:1 in the final LSTM model to detect the possibility of overfitting. The number of LSTM units for the final model was 256, with a batch size of 16. The epochs of the preliminary and final model were 50 and 100, respectively. Both models adopted adam as the optimizer and the mean absolute error was used as the loss function. Dataset of the preliminary and final models are presented in Table 2.

**Table 2:** Details of the dataset for the preliminary and final LSTM models.

	**Training dataset**	**Validation** **dataset**	**Testing dataset**	**Total**
**Preliminary model**				
Discharged in stable condition	15 (34.09%)	-	8 (18.18%)	23
Death	15 (34.09%)	-	6 (13.64%)	21
Total	30	-	14	44
**Final model**				
Discharged in stable condition	16 (36.36%)	4 (9.09%)	3 (6.82%)	23
Death	14 (31.82%)	4 (9.09%)	3 (6.82%)	21
Total	30	8	6	44

### Training of LSTM Survival Prediction Model

**Figure 4:**
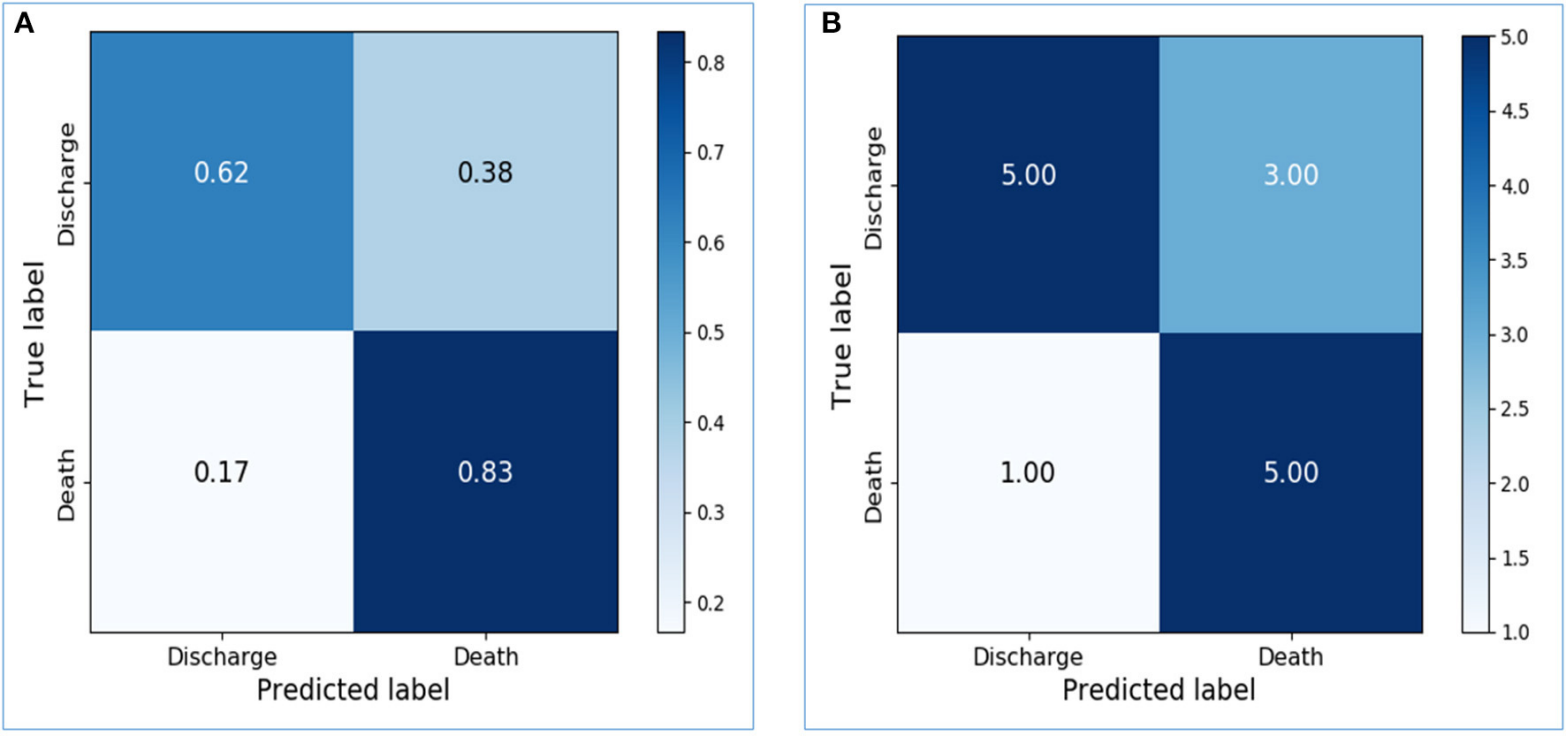
Confusion matrices of the preliminary prediction model. **(A)**: Confusion matrix of the testing dataset, with normalization. **(B)**: Confusion matrix of the testing dataset, without normalization.

Based on the activity data recorded in the initial 48 h after admission, the preliminary model yielded an accuracy of 0.8667 in the training dataset and 0.7143 in the testing dataset. The confusion matrix visualized the differences between model prediction and the ground truth. The variables used for the original and normalized confusion matrices were the same. In the normalized form of confusion matrices, the sum of each row is 1.0 and represents the correct prediction in terms of probability. Figures 4A,B illustrate the confusion matrices with normalization and without normalization, respectively. The sensitivity, specificity, PPV, NPV, and AUC of the model on the testing dataset were 0.8333, 0.625, 0.625, 0.8333, and 0.7292, respectively. These satisfactory results indicated the feasibility of LSTM in classifying time series data collected by wearable actigraphy devices without any physiological information.

**Figure 5:**
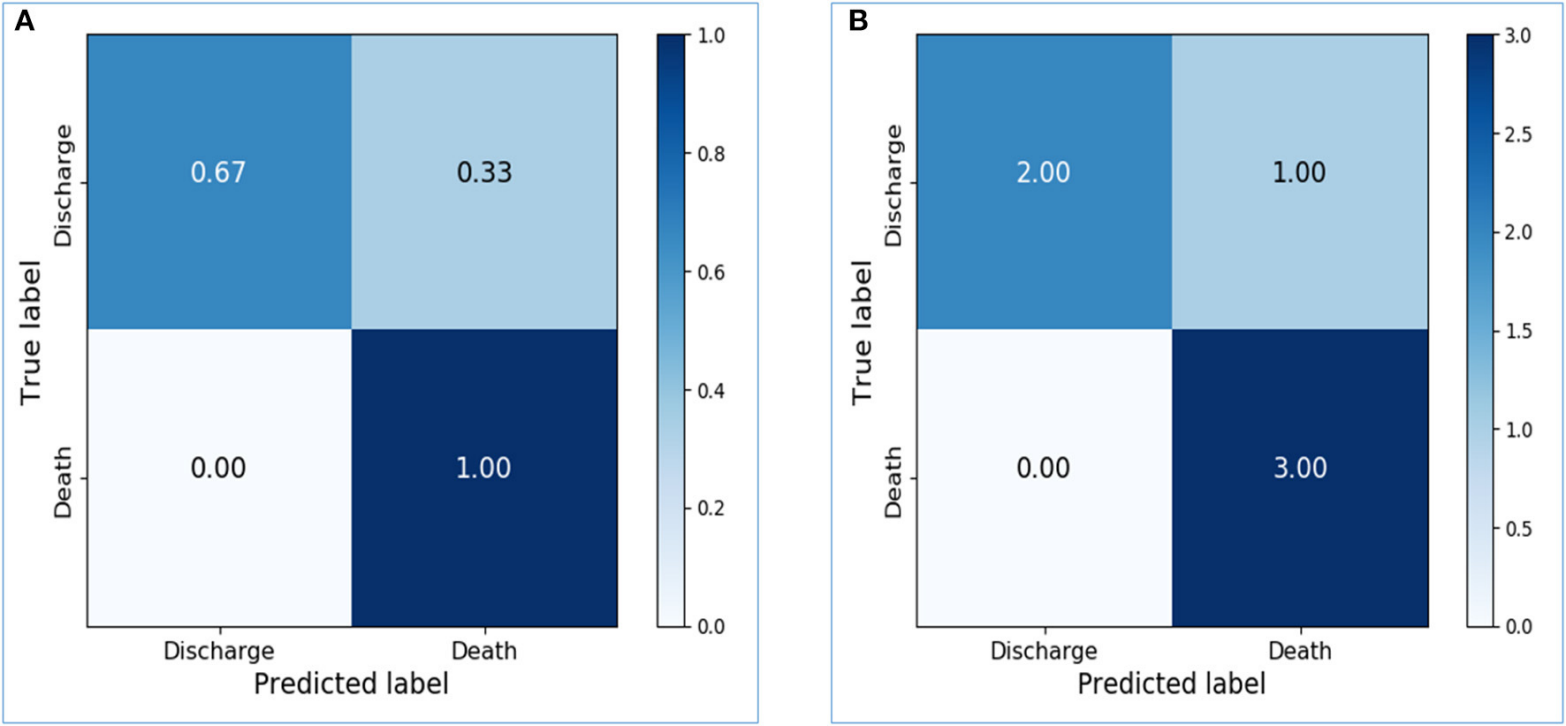
Confusion matrices of the final prediction model. **(A)** Confusion matrix of the testing dataset, with normalization. **(B)** Confusion matrix of the testing dataset, without normalization.

The dataset was further sliced into training, validation, and testing data in the final model with appropriate parameters. The training accuracy increased to 0.9667, and the validation accuracy and testing accuracy were 0.75 and 0.8333, respectively. After increasing the LSTM units from 64 to 256, the performance of the model on the testing dataset was greatly improved. Confusion matrices of the final model were shown in Figures 5A,B. The sensitivity, specificity, PPV, NPV, and AUC of the model on the testing dataset were 1.0, 0.6667, 0.75, 1.0, and 0.8333, respectively.

### The Impact of Data Length on LSTM Model Performance

Since activity data of the initial 48 h yielded favorable results, we further explored the performance of the model based on a shorter time series. The input data of the preliminary model and the final model were reduced from 48 to 24 h with the same parameters. The maximum length of data after zero-padding is 6,460. After reducing the time interval, the prognostic accuracy of both preliminary and final models decreased. The comparison of model performance based on 48 and 24 h is demonstrated in Table 3. The finding indicated decreasing classification accuracy of the models with reducing time length of the input data.

**Table 3:** Model performance with different input data lengths.

**Model**	**Training ACC^a^**	**Validation ACC**	**Testing ACC**	**Sensitivity**	**Specificity**	**PPV^b^**	**NPV^c^**	**AUC^d^**
Preliminary model 48 h	0.8667	N/A	0.7143	0.8333	0.625	0.625	0.8333	0.7292
Preliminary model 24 h	0.8333	N/A	0.6429	0.6667	0.625	0.5714	0.7143	0.6458
Final model 48 h	0.9667	0.75	0.8333	1.0	0.6667	0.75	1.0	0.8333
Final model 24 h	0.9333	0.625	0.6667	0.6667	0.6667	0.6667	0.6667	0.6667

a *ACC: accuracy.*

b *PPV: positive predictive value.*

c *NPV: negative predictive value.*

d *AUC: area under the receiver operating characteristic curve*.

## Discussion

The study proposed and examined the use of a wearable actigraphy device for survival prediction among end-stage cancer patients. Compared to the subjective PS evaluation by KPS, our results indicated that objective activity data recorded by the wearable devices also provided favorable prognostic accuracy when employing the LSTM model. The wearable actigraphy device employed in this study is a lightweight and low-cost device, and based on the results, provides convenient activity data for survival prediction in end-stage cancer patients. The findings of this study suggest implementing the wearable technology and the survival prediction model in end-of-life care to facilitate decision-making for clinicians and better preparation for patients and their families.

PS evaluation can inform patients' clinical condition and treatment decisions in end-of-life care. However, subjective evaluation tools like KPS are seldomly used as a single predictor for patient survival; one of the reasons is the potential risk of measurement bias due to their subjective nature (37). As a result, studies examining the applicability of objective activity evaluation, such as measurements by wearable technology, are being conducted to investigate the usability of activity data for survival prediction. While several studies have identified associations between activity data of cancer patients and their clinical outcomes, such as unplanned healthcare encounters (38), adverse events, hospitalizations, and survival (25), no previous studies have utilized the activity data to build a prediction model that suggests survival outcomes. To our knowledge, this is the first study that applied objective activity data of patients in a deep-learning model to provide survival outcome predictions in the end-stage cancer population.

In this study, while KPS had comparable performance, PPI yielded a nearly impeccable result regarding prognostic accuracy. However, it should be noted that the accuracy of these prognostic tools, either KPS or PPI, relies heavily on the judgment of an experienced clinical practitioner. In comparison, the activity monitoring and survival prediction model proposed by this study, requires no clinical expertise but a wearable wristband. The advantage introduces two clinical implications: first, automatically-generated survival predictions can lessen healthcare practitioners' workload in clinical settings, and second, enable end-of-life care at places outside hospitals, such as hospice at home. The result also suggested that integrating activity evaluation and clinical parameters in a survival prediction model might facilitate better prognostic accuracy, and subsequent analysis should be conducted to investigate the feasibility of such a combination.

The activity data of only the initial 24 and 48 h since patients' hospital admission was employed to provide timely survival prediction and enable practicable use in the clinical settings. Activity recordings fewer than 24 h were not analyzed due to the consideration of circadian rhythm (39). Circadian rhythms are part of the body's internal clock and are approximately 24 h a cycle. However, studies have shown that circadian rhythms can be disrupted by multiple factors, including the states of cancerous diseases (40); thus, we employed activity analysis of both 24 and 48 h to include at least a cycle of the circadian rhythm. Our findings showed that the predictions based on activity data of 48 h yielded better prognostic accuracy than 24 h in both preliminary and final models. While the better performance of the model may be attributed to the increasing length of data (41), the inclusion of at least a cycle of circadian rhythms might also serve as a constructive factor. Future studies examining the impact of circadian rhythm on activity data of end-stage cancer patients are thus warranted.

Though the study offers promising results of the deep-learning-based survival prediction model, the study still encompasses a few limitations. First, the issue of data discontinuity was noticeable. Probable causes include battery charging requirements and the non-waterproof characteristics of the device, as these monitors were removed during the showering time. Although the issue of data discontinuity and different data lengths were handled by data pre-processing, future studies with better activity tracking devices and data quality are warranted. Second, the study was designed to provide patients' outcomes at the end of their hospital stay, either death or discharged in stable condition. Even though the survival time varied among participants regardless of their final survival outcomes, the proposed model only informed binary survival outcomes rather than the estimated survival time. Finally, we failed to adopt PPI assessments at the beginning of the study and thus, only applied the tool to the last 20 participants.

In conclusion, the study presented a wearable activity monitoring and survival prediction model for end-stage cancer patients in hospice care settings. Our survival prediction model provided satisfactory prognostic accuracy of patients' binary survival outcomes, death or discharged in stable condition, by using activity data of the initial 24 or 48 h on their hospital admission. The prognostic accuracy of the model was time-dependent, with models using activity data of 48 h yielding better results than those of 24 h. The automatically-generated survival prediction by the LSTM deep-learning model demonstrated feasibility in clinical settings and may benefit end-of-life care in settings without healthcare professionals.

## Data Availability Statement

The raw data supporting the conclusions of this article will be made available by the authors, without undue reservation.

## Ethics Statement

The studies involving human participants were reviewed and approved by Taipei Medical University-Joint Institutional Review Board. The patients/participants provided their written informed consent to participate in this study.

## Author Contributions

TY, YH, SM, L-SL, and SS-A conceived of the presented idea. YH, H-WL, L-SL, and J-FC were in charge of clinical evaluations and data collection. TY and SM performed the statistical analysis of clinical parameters. P-YK and L-WT performed data-processing and deep-learning prediction model building. L-SL and C-WS validated the analytical methods. SS-A, C-WS, and J-FC co-supervised the project. All authors discussed the results and contributed to the final manuscript.

## Funding

This work was supported in part by Ministry of Science and Technology, Taiwan [Grant Numbers 108-2221-E-038-013, 110-2923-E-038-001-MY3, 110-5420-003-300, 110-2320-B-038-056, 109-2221-E-009-018-MY3, 109-2314-B-038-122, 109-2314-B-038-141, 109-2635-B-038-001, and 109-2314-B-038-072], Taipei Medical University, Taiwan [Grant Numbers 108-3805-009-110 and 109-3800-020-400], Ministry of Education, Taiwan [Grant Number 108-6604-002-400], and Wanfang hospital, Taiwan [Grant Number 106TMU-WFH-01-4].

## Conflict of Interest

The authors declare that the research was conducted in the absence of any commercial or financial relationships that could be construed as a potential conflict of interest.

## Publisher's Note

All claims expressed in this article are solely those of the authors and do not necessarily represent those of their affiliated organizations, or those of the publisher, the editors and the reviewers. Any product that may be evaluated in this article, or claim that may be made by its manufacturer, is not guaranteed or endorsed by the publisher.
